# Supplementary material for: Happiness and the Patterns of Life: A Study of Geolocated Tweets
Source: Sci Rep. 2013 Sep 12;3:2625. doi: 10.1038/srep02625 (PMC6505625; doi:10.1038/srep02625)
Supplement: Supplementary Information — Supplementary Materials [file srep02625-s1.pdf]

Supplementary Material:
Happiness and the Patterns of Life: A Study of Geolocated Tweets

Morgan R. Frank, Lewis Mitchell, Peter S. Dodds, Christopher M. Danforth

Computational Story Lab, Department of Mathematics and Statistics, Vermont Complex Systems Center, Vermont Advanced Computing Core, University of Vermont, Burlington, Vermont, United States of America

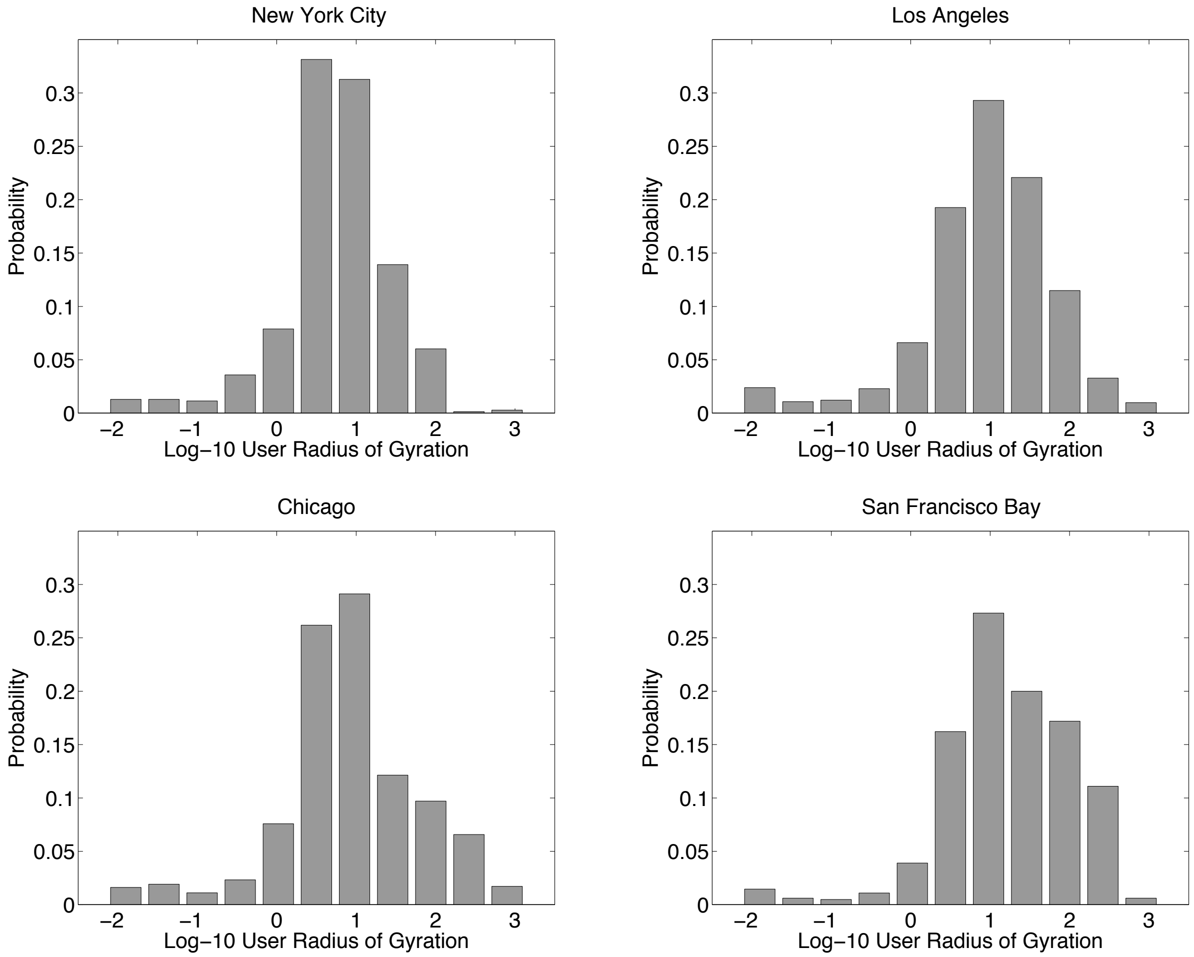

Figure S1: The distributions of gyradius (km) for four cities appear to be approximately lognormal. The mode distance (binned) is larger for Los Angeles and San Francisco than for Chicago and New York City. We note that these distributions were calculated for all individuals whose expected location fell within the latitude and longitude bounds of main text Fig. 2, and thus reflect a modified set of individuals than those identified with cities in Fig. S3.

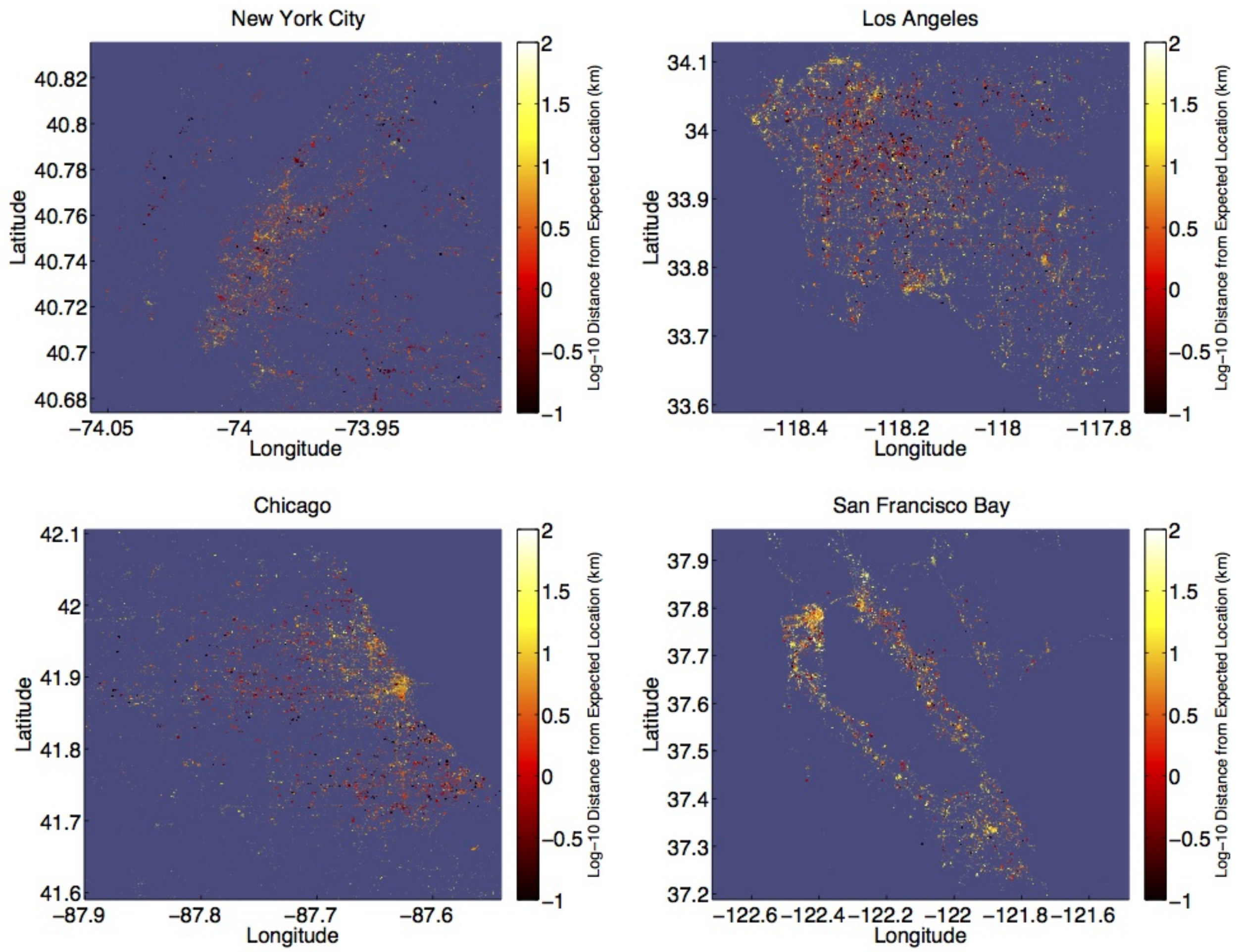

Figure S2: The distance from expected location, calculated for each individual, is shown for each tweet authored in four example cities in 2011. Spatial clustering is observed (Table S2); messages authored by individuals far from their expected location are more likely to appear close to each other. The number of tweets shown for each city is $N = 56,650$ (Chicago), $N = 103,213$ (Los Angeles), $N = 42,089$ (New York City), and $N = 45,754$ (San Francisco).

City	Geary's C (p-value)	Moran's I (p-value)	Tweets
Chicago	0.43 ($< 10^{-15}$)	0.27 ($< 10^{-15}$)	56650
Los Angeles	0.47 ($< 10^{-15}$)	0.22 ($< 10^{-15}$)	103213
New York City	0.40 ($< 10^{-15}$)	0.34 ($< 10^{-15}$)	42089
San Francisco Bay	0.37 ($< 10^{-15}$)	0.46 ($< 10^{-15}$)	45754

Table S1: Evidence for clustering is observed in both Geary's C and Moran's I spatial autocorrelation for tweet location colored by gyradius (Figure 2).

City	Geary's C (p-value)	Moran's I (p-value)	Tweets
Chicago	0.74 ($< 10^{-15}$)	0.14 ($< 10^{-15}$)	56650
Los Angeles	0.64 ($< 10^{-15}$)	0.16 ($< 10^{-15}$)	103213
New York City	0.65 (1.3×10^{-3})	0.07 ($< 10^{-15}$)	42089
San Francisco Bay	0.55 ($< 10^{-15}$)	0.34 ($< 10^{-15}$)	45754

Table S2: Evidence for clustering is observed in Geary's C and Moran's I spatial autocorrelation for tweet distance from expected location as well (Figure S2).

City	Geary's C (p-value)	Moran's I (p-value)	Individuals
Chicago	0.43 ($< 10^{-15}$)	0.60 ($< 10^{-15}$)	563
Los Angeles	0.29 ($< 10^{-15}$)	0.70 ($< 10^{-15}$)	983
New York City	0.21 ($< 10^{-15}$)	0.75 ($< 10^{-15}$)	387
San Francisco Bay	0.52 (2.3×10^{-12})	0.44 ($< 10^{-15}$)	423

Table S3: Evidence for clustering is observed in Geary's C (local) and Moran's I (global) spatial autocorrelation calculated for mode location colored by gyradius (not shown to preserve privacy). Note that Geary's C values fall between 0 and 2, with 1 indicating no correlation and values smaller than 1 suggesting increasing correlation. Moran's I values range from -1 to 1, with larger values suggesting positive correlation.

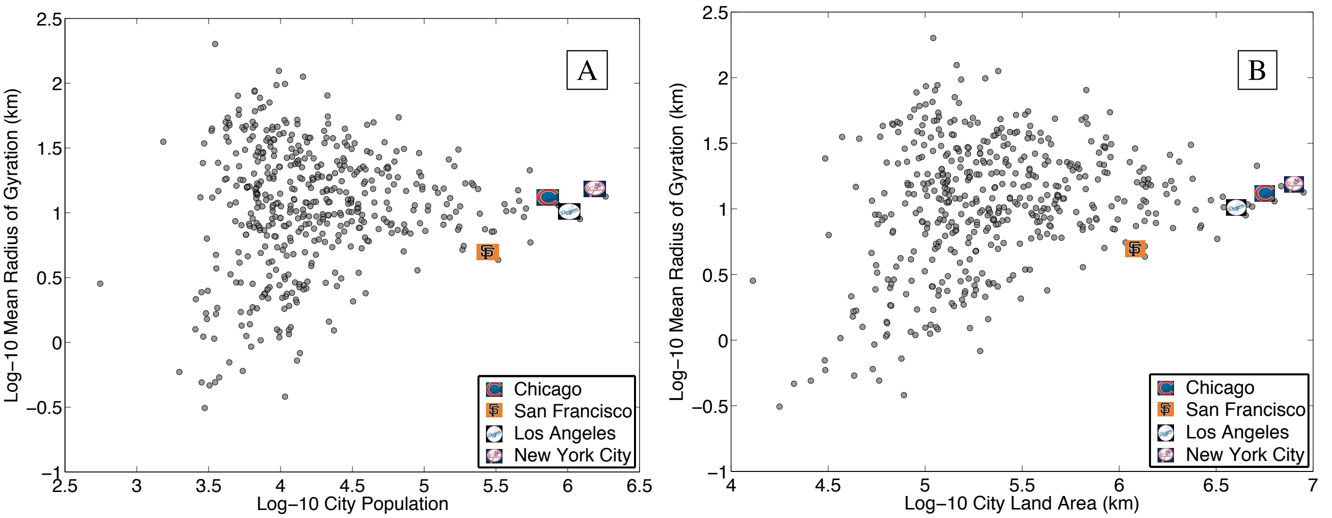

Figure S3: The mean gyradius of individuals whose expected location falls within each city is plotted against the city's population (A) and land area (B). Shown are cities containing at least 50 individuals with a nonzero gyradius, each individual having authored at least 30 geolocated tweets. City boundaries are defined by [2] which encompasses a smaller area for the four cities illustrated in the main text Fig. 2. Generally, gyradius increases with city population and land area, with no large cities exhibiting a small mean radius. Pearson correlations: Population $\rho = 0.10,\ p = 0.03$, Land Area $\rho = 0.24,\ p = 2 \times 10^{-7}$.

rank	radius (km)	city
1	200.6	Martinsville, VA
2	124.5	Middletown, OH
3	112.3	Elkhart, IN
4	98.8	Pottstown, PA
5	96.6	Decatur, IL
…	…	…
215	13.3	New York City, NY
247	11.4	Chicago, IL
300	8.94	Los Angeles, CA
387	4.33	San Francisco & Oakland, CA
…	…	…
468	0.492	Greenville, MS
469	0.491	Athens, OH
470	0.465	Key West, FL
471	0.381	El Centro Calexico, CA
472	0.312	Pullman, WA

Table S4: Top and Bottom 5 cities with respect to mean gyradius, along with the four cities investigated in main text Fig 2.

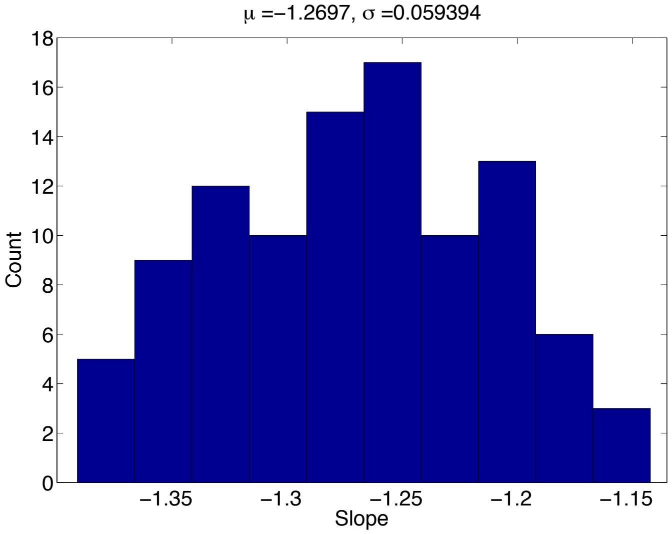

Figure S4: A random 10% of individuals (30 out of 300) are removed from Figure 5A, and the slope of the probability fit (red curve) is recalculated. Repeating the procedure 100 times, we find the above distribution of slopes. The mean of this distribution agrees well with that reported in Figure 5A. Additionally, fitting the power law model to the leading 10 locales, using only individuals who have at least 10 locales, we also get a slope of roughly -1.3 (not shown).

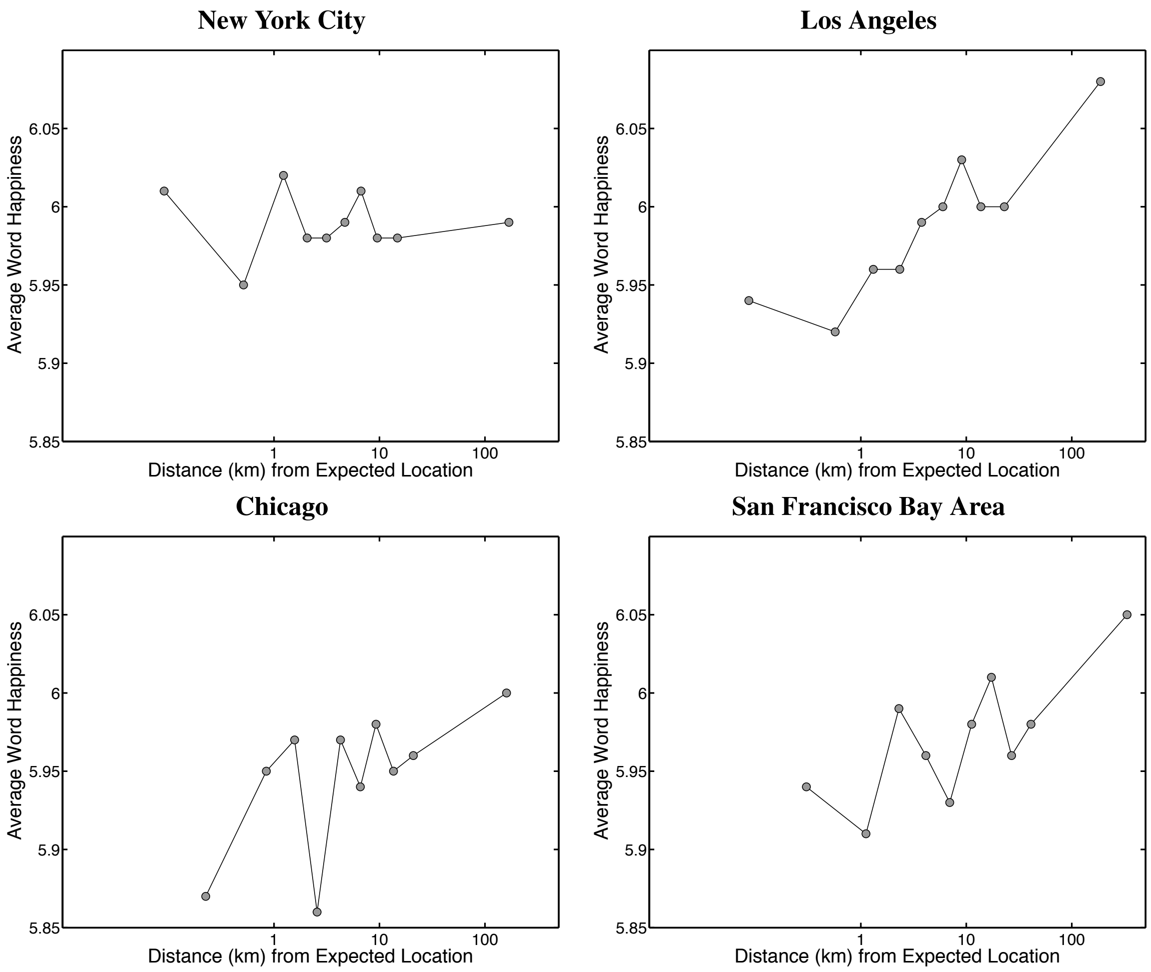

Figure S5: For New York City, Los Angeles, Chicago, and the San Francisco Bay Area, we group messages into equally sized bins by the distance from expected location of their author, and measure the average word happiness of each group. These plots exhibit similar trends to that observed in main text Fig. 6A with the exception of New York City.

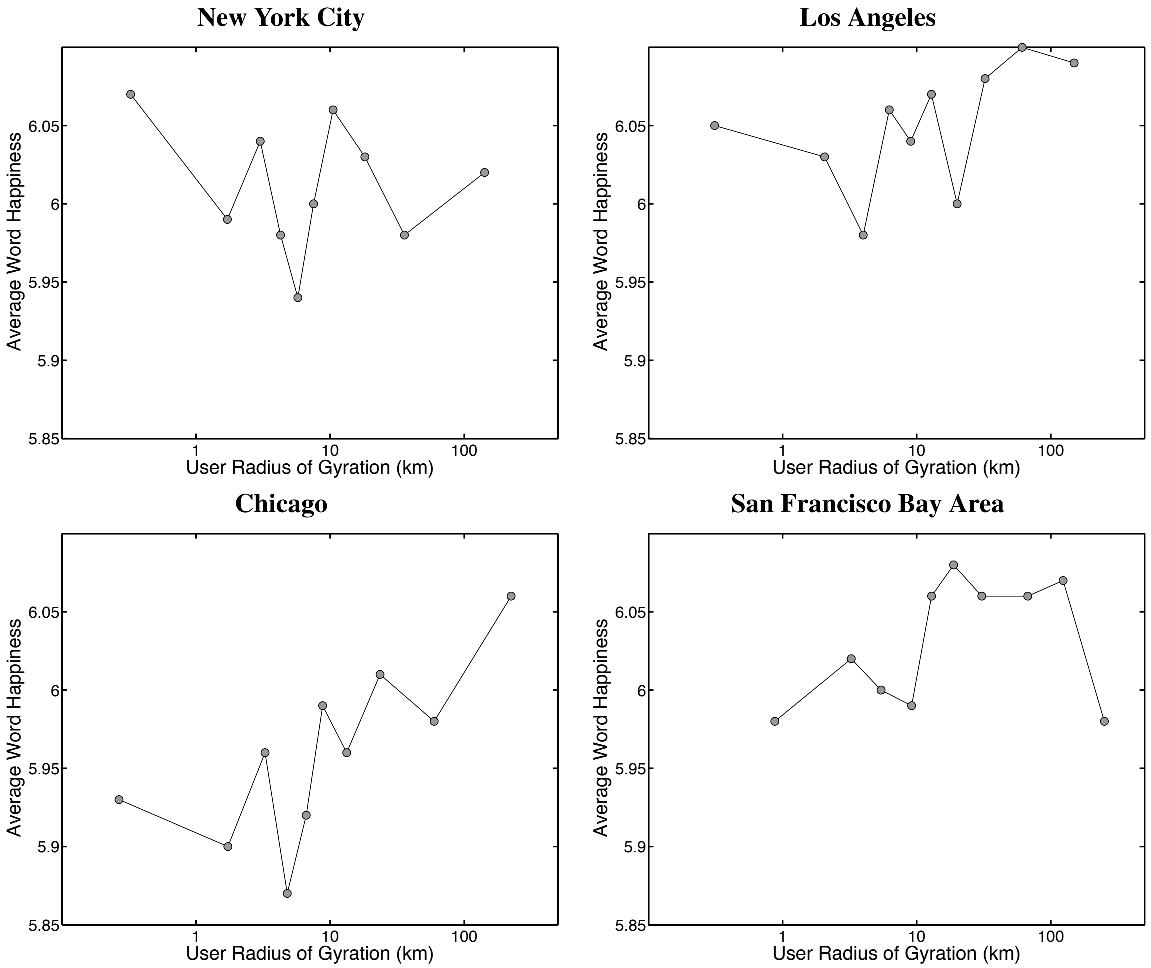

Figure S6: For New York City (130 individuals/bin), Los Angeles (175 individuals/bin), Chicago (125 individuals/bin), and the San Francisco Bay Area (63 individuals/bin), we group individuals into equally sized bins by their gyradius and measure the average word happiness of each group. These plots exhibit similar trends to that observed in main text Fig. 6B with the exception of the largest radius group in the San Francisco Bay Area, and New York City as a whole.

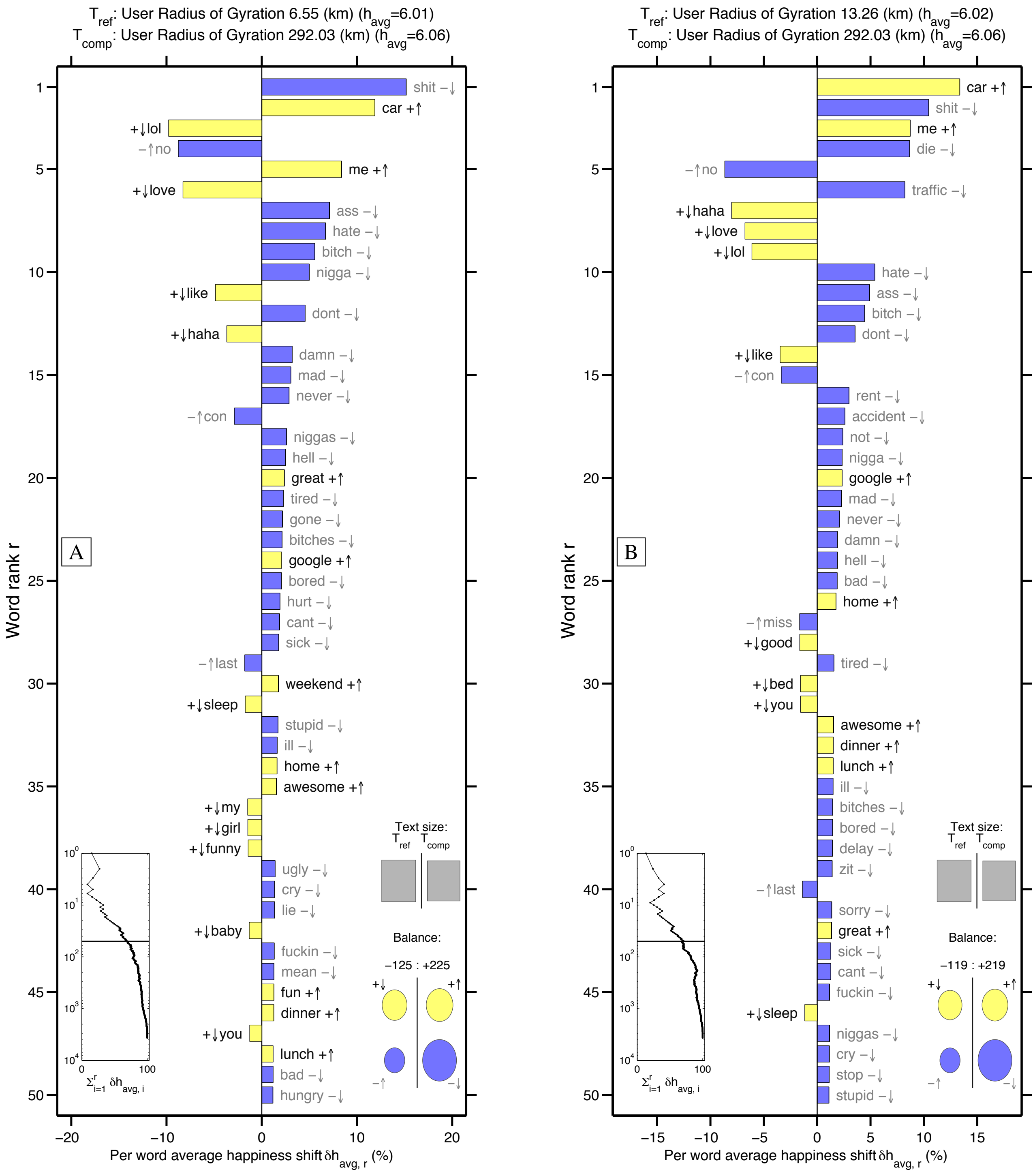

Figure S7: We compare the 6.55 km gyradius group versus the 292.03 km gyradius group (A). We find that the 292.03 km group has relatively frequent use of the words ‘car’ and ‘weekend’ suggesting that this group travels on the weekends perhaps to a vacation home as suggested by use of the word ‘home’. (B) We compare the 13.26 km gyradius group versus the 292.03 km gyradius group. We find that the 292.03 km group uses the word ‘car’ more frequently than the 13.26 km group which, interestingly, uses the word ‘traffic’ more frequently. Again the increased relative usage of these words seems fitting for a groups with these patterns of movement.

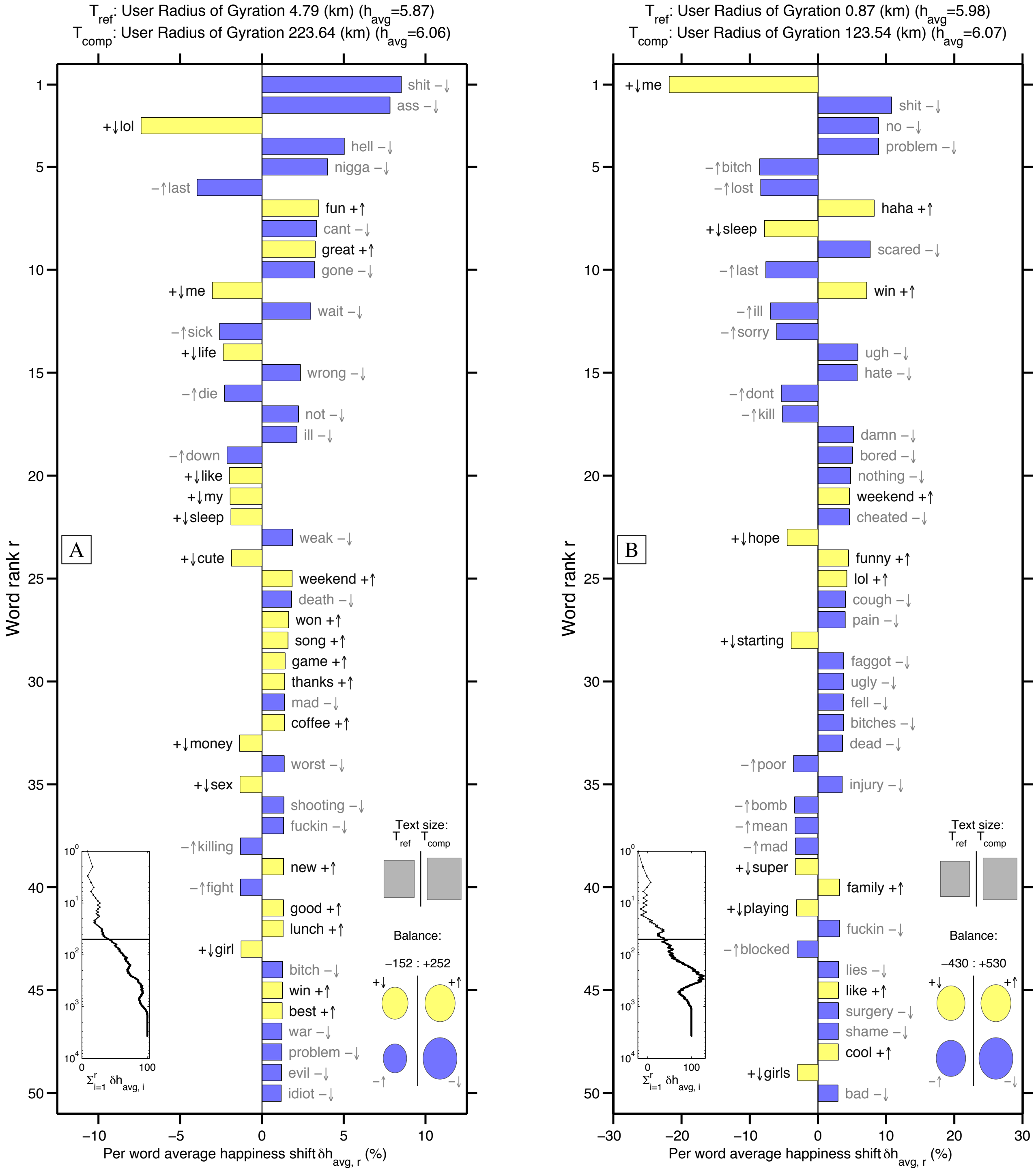

Figure S8: (A) A word shift comparing the 4.79 km gyradius group to the 223 km gyradius for Chicago. We observe the first group is less happy because of increased usage of profanity and negative words like ‘can’t’, ‘gone’, and ’wrong’. (B) A word shift comparing the .87 km gyradius group to the 123.54 km gyradius group for the San Francisco Bay Area. We find the second group to be happier because of an increase in positive words like ‘haha’, ‘win’, ‘weekend’, ‘funny’, and ‘lol’, along with a decrease in negative words like ‘no’, ‘problem’, and ‘hate’.

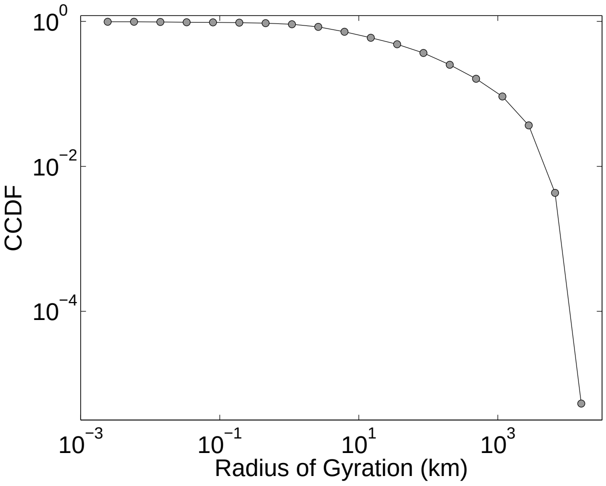

Figure S9: Complementary Cumulative Distribution Function (CCDF) for the gyradii of all users with at least 30 geotagged messages. Gonzalez [1] found this distribution to be well modeled by a truncated power law with an exponential tail.

Normalizing Human Trajectory

To compare the shape of trajectories of individuals traveling in different directions and over different distances, we use the methods introduced by González et al. [1]. We will examine the normalization steps for two individuals we will call user A and user B. We have 768 geolocated tweets for user A and 1,882 geolocated tweets for user B. User A has gyradius $r^A = 463.61$ km and user B has gyradius $r^B = 54.28$ km. Fig. S10 represents the geospatial tweet locations for user A and user B, but we have shifted their coordinate system to maintain their anonymity. We have also allowed for a slight spatial separation between the locations for user A and the locations of user B for clarity.

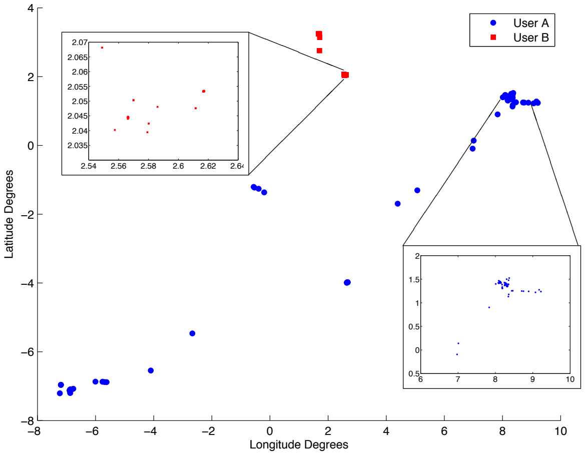

Figure S10: Tweet locations for User A and User B.

In Fig. S11, we apply the linear transformation shifting each location for the user to the distance in kilometers from their center of mass, i.e. the expected location of the user. The difference in gyradius between user A and user B is still very apparent in the axis ranges for this plot. Notice that the directional relationships between the tweet locations for each user have still been preserved. We can see that user A travels predominantly in a southwest direction, while user B travels primarily in a northwest direction.

To normalize for direction of travel, let the set of tweet locations for user i be represented by the set of equally weighted masses at each of the tweet locations $\{(x_1,y_1),(x_2,y_2),\ldots,(x_n,y_n)\}$. Now we calculate the tensor of inertia (I) for each set of weighted (x,y)-points as

$$I = \begin{bmatrix} \sum_{j=1}^{n} x_j^2 & -\sum_{j=1}^{n} x_j y_j \\ -\sum_{j=1}^{n} x_j y_j & \sum_{j=1}^{n} y_j^2 \end{bmatrix}$$

The eigenvector of I corresponding to the largest eigenvalue of I represents the direction along which most of user i's trajectory occurs; we call this the principal axis for user i (see Fig. S12).

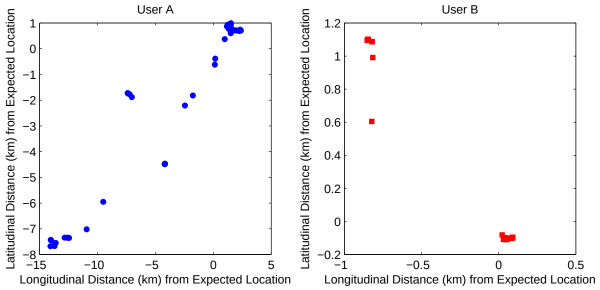

Figure S11: Tweet locations for User A and User B transformed to the distance in kilometers from their expected locations, respectively.

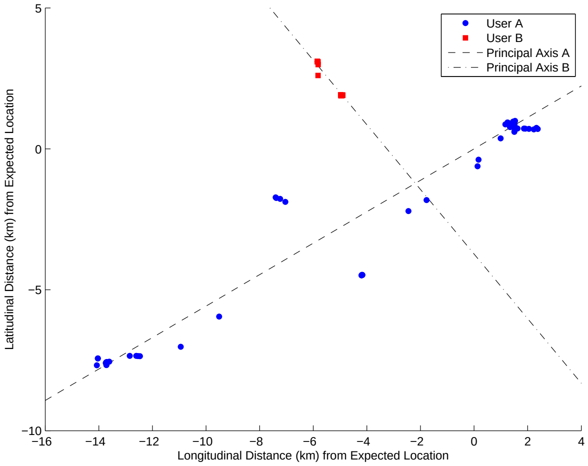

Figure S12: The tweet locations for User A and User B along with a line representing the principal axis for that user.

Now we can determine the angle necessary to rotate the set of points for user i so that the the resulting principal axis is the x-axis. Fig. S13 shows the results of this step. We see that the principal axis for user A and user B is now the x-axis.

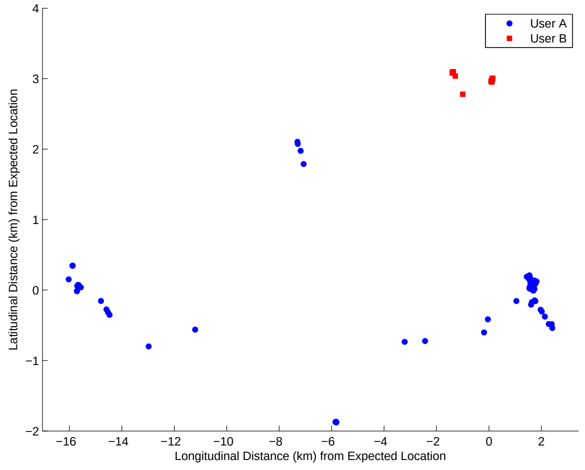

Figure S13: The results after rotating the locations of User A and User B. We see that they now both have principal axes of trajectory pointing due west.

The final step is to normalize for individuals with different gyradius. We accomplish this by dividing the x-coordinate of each rotated tweet location for user i by σ_x, where σ_x is the standard deviation of the x-coordinates of the rotated tweet locations for user i, and similarly dividing by σ_y for the y-coordinates. The final result is shown in Fig. S14.

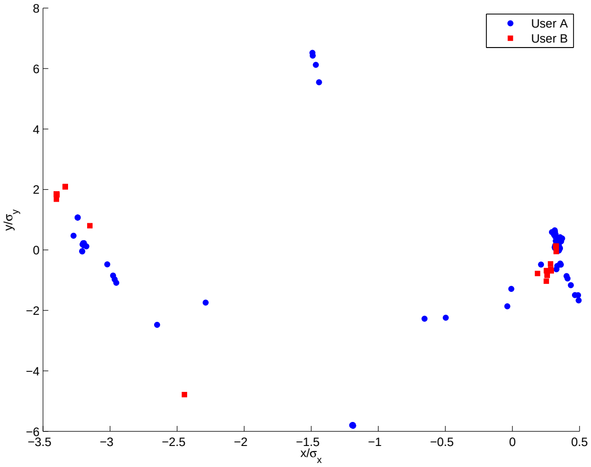

Figure S14: The rotated tweet locations of User A and User B after normalizing for gyradius. The origin represents the center of mass of the respective individuals' trajectory, namely $\langle \vec{p}^a \rangle$ from equation (2).

As a result, we can compare the shape of the trajectories for User A and User B having normalized for direction and gyradius. We can see that both User A and User B have most of their normalized tweet locations in two main clusters.

References

[1] González, M. C., Hidalgo, C. A., Barabási, A. L. Understanding individual human mobility patterns. *Nature*. Vol. 453, pp. 779-782, (2008) doi:10.1038/nature06958

[2] U.S. Census Bureau Geography Division. *2010 Census TIGER/Line Shapefiles*. http://www.census.gov/geo/www/tiger/tgrshp2010/tgrshp2010.html, accessed February 2013.
